# Synthetic Aminoindanes: A Summary of Existing Knowledge

**DOI:** 10.3389/fpsyt.2017.00236

**Published:** 2017-11-17

**Authors:** Nikola Pinterova, Rachel R. Horsley, Tomas Palenicek

**Affiliations:** ^1^National Institute of Mental Health, Klecany, Czechia; ^2^3rd Medical Faculty, Charles University, Prague, Czechia

**Keywords:** aminoindanes, 5,6-methylenedioxy-2-aminoindane, 5-iodo-2-aminoindane, 2-aminoindane, 5,6-methylenedioxy-*N*-methyl-2-aminoindane, 5-methoxy-6-methyl-2-aminoindane

## Abstract

**Objectives:**

Aminoindanes (“bath salts,” a class of novel psychoactive substances, NPSs) increased rapidly in popularity on the recreational drug market, particularly after mephedrone and other synthetic cathinones were banned in the UK in 2010. Novel aminoindanes continue to emerge, but relatively little is known about their effects and risks. Their history, chemistry, pharmacology, behavioral effects, pharmacokinetics, and toxicity are reviewed in this paper.

**Methods:**

Scientific literature was searched on ISI Web of Knowledge: Web of Science (WoS) during June and July 2017, using English language terms: aminoindanes such as 5,6-methylenedioxy-2-aminoindane (MDAI), 5-iodo-2-aminoindane (5-IAI), 2-aminoindane (2-AI), 5,6-methylenedioxy-*N*-methyl-2-aminoindane (MDMAI), and 5-methoxy-6-methyl-2-aminoindane (MMAI). WoS was selected as it searches several databases simultaneously and has quality criteria for inclusion. For typical use and effects, Erowid, PsychonautWiki, Bluelight, and Drugs-Forum were searched; for legal status and epidemiology, the European Information System and Database on New Drugs (EDND) was used.

**Results:**

Aminoindanes were first synthesized for medical use, e.g., as anti-Parkinsonian drugs and later as a potential compound facilitating psychotherapy; however, they are now widely substituted for ecstasy. Their mechanisms of action (primarily *via* serotonin) mean that they may pose a significant risk of serotonin syndrome at high doses or when combined with other drugs. Fatally toxic effects have been observed both in the laboratory in animal studies and in clinic, where deaths related with aminoindanes have been reported.

**Conclusion:**

Greater knowledge about aminoindanes is urgently required to decrease risks of fatal intoxication, and appropriate legislation is needed to protect public health without impeding research.

## Introduction

During the past decade, there has been a dramatic increase in the number and variety of novel psychoactive substances (NPSs) available on the illicit and gray drug markets (particularly *via* the Internet and “dark web”). In 2014, the number of NPSs boomed with 101 new compounds detected. In 2016, approximately one new NPS per week was identified, and the European Monitoring Centre for Drugs and Drug Addiction (EMCDDA) was monitoring more than 620 NPSs (1). One of the first NPSs that became widely used recreationally was the cathinone derivative mephedrone (4-MMC, 4-methylmethcathinone), marketed at the time as a “legal” substitute for ecstasy (MDMA, 3,4-methylenedioxymethamphetamine) and cocaine, sharing effects of both (2). Mephedrone and other cathinones such as methylone (βk-MDMA, 3,4-methylenedioxy*-N-*methcathinone) and butylone (βk-MBDB, β-keto*-N-*methylbenzodioxolylbutanamine) were initially sold as, e.g., “bath salts” or “plant food,” labeled “not for human consumption.” Mephedrone became very popular due to its low price, high purity, and “legality” and, in the UK, it rapidly became as widespread as cocaine (3). In 2009 and 2010, the UK government placed piperazine derivatives, mephedrone, and other related cathinones under legal control (4), which resulted in their immediate replacement with new structural analogs and with a new class of NPSs: synthetic aminoindanes. One of the first was 5,6-methylenedioxy-2-aminoindane (MDAI), which claimed to be a “legal,” non-neurotoxic analog of MDMA, with strong empathogenic and weaker stimulatory effects (5). Aminoindanes such as MDAI, 5,6-methylenedioxy*-N-*methyl-2-aminoindane (MDMAI), 5-iodo-2-aminoindane (5-IAI), 2-aminoindane (2-AI), 5-methoxy-6-methyl-2-aminoindane (MMAI), and 5-methoxy-2-aminoindane (MEAI) represent a relatively new generation of NPS. Cases of acute toxicity, including fatal poisoning, have been reported with their use (6). Only minimal reliable information on aminoindanes exists at present and, owing to their increasing popularity, the present brief review is timely. The paper summarizes the history of their creation, therapeutic potential in medical research and subsequent discovery by recreational drug users, their pharmacology, behavioral effects, pharmacokinetics, and toxicity.

## Method

**Figure 1:**
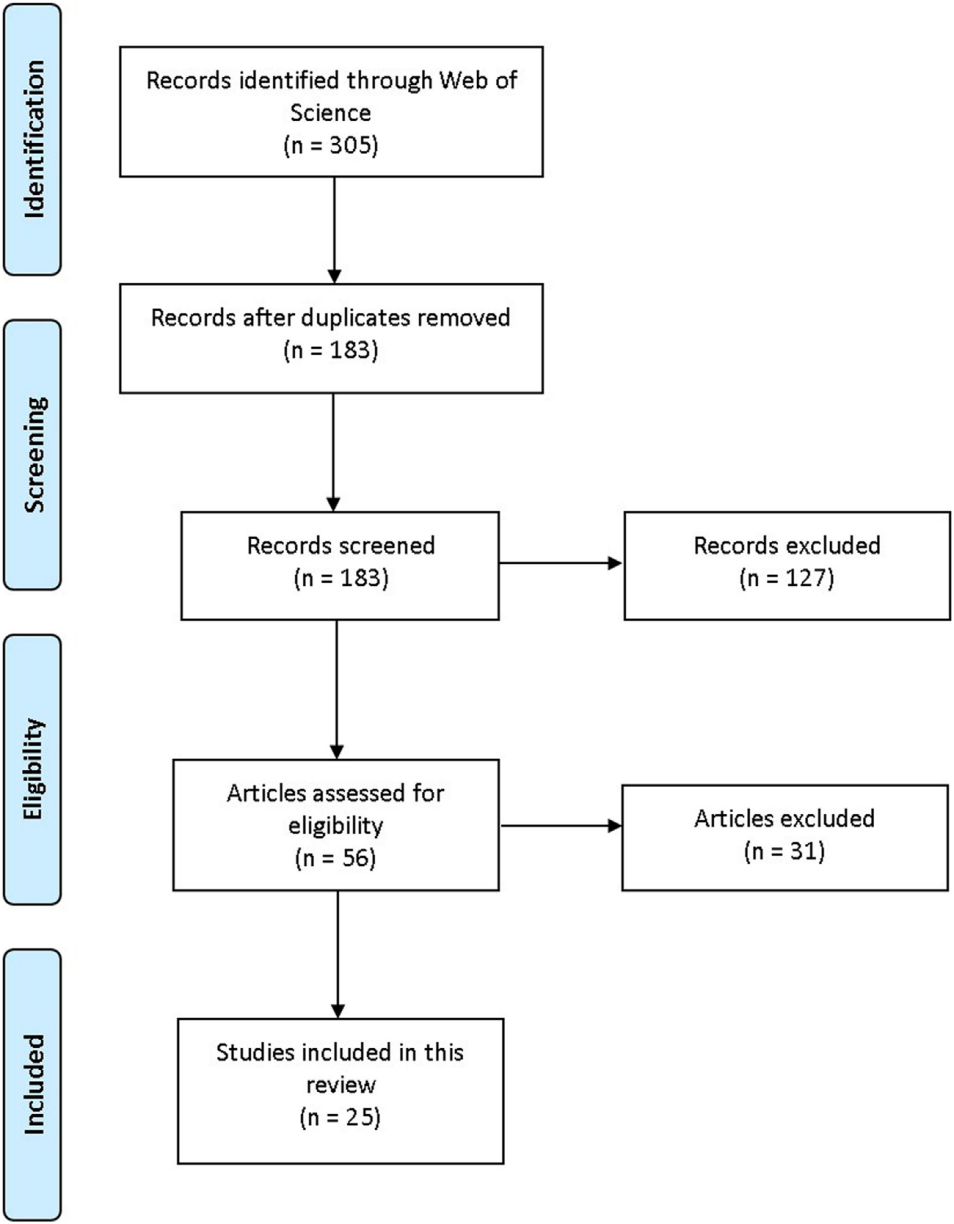
PRISMA flowchart visualization of the search and selection process. Adapted from Moher et al. (7).

ISI Web of Knowledge: Web of Science (WoS) was searched during June and July 2017. WoS was selected because it simultaneously searches other databases such as PubMed and ScienceDirect, and includes quality criteria for inclusion (e.g., peer review). Keywords (used separately and in combination) were as follows: aminoindane, MDAI, 5,6-methylenedioxy-2-aminoindane, 5-IAI, 5-Iodo-2-aminoindane, 2-aminoindane, MDMAI, 5,6-Methylenedioxy-N-methyl-2-aminoindane, MMAI, 5-Methoxy-6-methyl-2-aminoindane. Full empirical/review articles containing relevant information about aminoindanes written in the English language were included; no date limits applied (Figure 1). When suitable articles were found, citation searches were also conducted. For subjective effects, typical use and doses, Erowid,^1^ PsychonautWiki,^2^ Bluelight,^3^ and Drugs-Forum^4^ (Internet discussion fora and wikis) were searched using the same search terms as above. Information about legal status and availability of aminoindanes in the European Union (EU), the EDND was consulted *via* the senior author, Dr. Palenicek, through the “Working group: Early warning system on new drugs,” National Monitoring Centre for Drugs and Addiction, Czech Republic.

## Results

### Chemistry

2-aminoindane is an amphetamine (AMPH) analog with a rigid conformation due to a bridge between the α-carbon and the aromatic ring (8). In the 1990s, Nichols et al. synthesized cyclic analogs of 3,4-methylenedioxyamphetamine (MDA), MDMA, 3-Methoxy-4-methylamphetamine (MMA), and *p*-iodoamphetamine (PIA) containing the 2-AI compound. Their procedures for synthesizing aminoindanes were well described (9–13). NPSs synthesized from the substances listed above are MDAI, MDMAI, MMAI, and 5-IAI (Table 1); all of these are psychoactive and their presence on the market has been confirmed in confiscated samples of “legal highs” (14). The EU Early Warning System^5^ and the United Nations Office on Drugs and Crime (UNODC) Early Warning Advisory (EWA) on New Psychoactive Substances^6^ have reported additional novel substances with an aminoindane structure, such as NM-2AI (*N*-methyl-2-aminoindane), 1-AI (1-aminoindane), and a fenfluramine analog ETAI (*N*-ethyl-5-trifluoromethyl-2-aminoindane); however, there is currently no scientific information available about these compounds.

**Table 1:** Chemical structures and names, International Union of Pure and Applied Chemistry (IUPAC) names of amphetamine, MDMA, MDA, 2-aminoindane, and its derivatives with psychoactive effects.

Structure	Name	Chemical name	lUPAC name
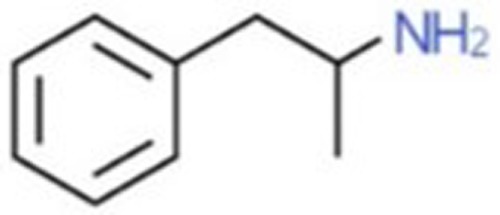	Amphetamine, AMPH, Speed	Alpha-methylphenethylamine	l-phenylpropan-2-amine
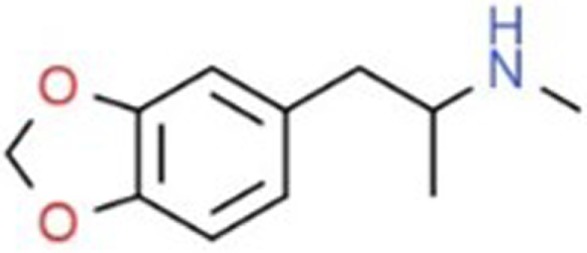	MDMA, Ecstasy, Molly, X, XTC	3,4-methylenedioxymethamphetamine	l-(1,3-benzodioxol-5-yl)*-N-*methylpropan-2-amine
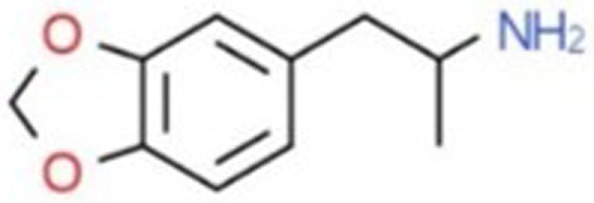	MDA	3,4-methylenedioxyamphetamine	l-(1,3-benzodioxol-5-yl)propan-2-amine
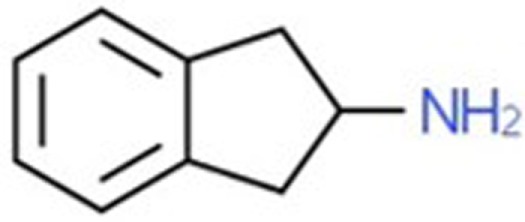	2-AI	2-aminoindane	2,3-dihydro-1H-inden-2-amine
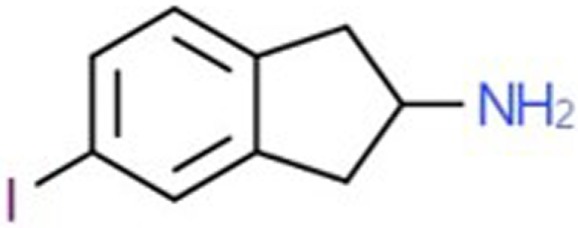	5-IAI	5-Iodo-2-aminoindane	5-iodo-2,3-dihydro-1H-inden-2-amine
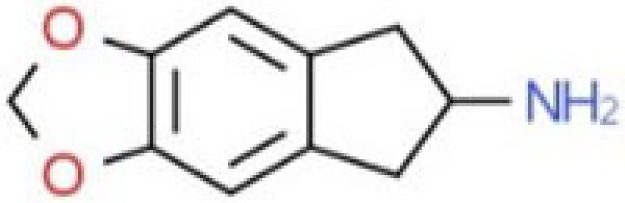	MDAI	5,6-methylenedioxy-2-aminoindane	6,7-dihydro-5H-cyclopenta[f][1,3]benzodioxol-6-amine
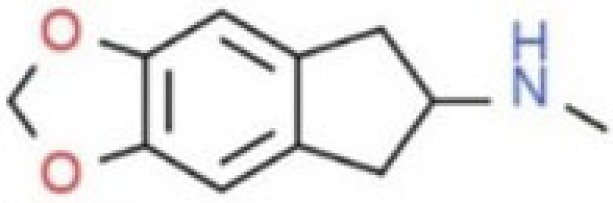	MDMAI	5,6-methylenedioxy-2-methylaminoindane	N-methyl-6,7-dihydro-5H-cyclopenta[f][1,3]benzodioxol-6-amine
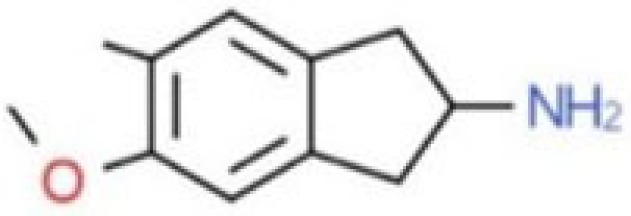	MMAI	5-methoxy-6-methyl-2-aminoindane	5-methoxy-6-methyl-2,3-dihydro-1H-inden-2-amine

### Origins of Aminoindanes in Pharmacological Research

Owing to an amino group, aminoindanes are potentially vasoactive and bronchodilatory, which was the main focus for their initial development (15, 16). Since the chemical structure of aminoindanes is similar to that of AMPHs (owing to the presence of the phenethylamine skeleton), there was a strong assumption that aminoindanes would have the same bronchodilatory effect as ephedrine. Therefore, Levin et al. (17) evaluated the bronchodilatory and toxic effects of 2-AI and its N-substituted derivatives in the rat. 2-AI hydrochloride given intravenously showed less toxicity than AMPH hydrochloride, and 2-AI derivatives were more effective bronchodilators as compared with L-ephedrine. Aminoindanes have also been studied for their analgesic potency (comparable to morphine sulfate)—potency to increase blood pressure, respiration, and spinal reflexes (18, 19).

Based on Kier’s receptor mapping technique (20)—a drug discovery method where the distance between oxotremorines’s heteroatoms and dopamine’s heteroatoms in reported conformations is similar—Martin et al. (21) designed and synthesized a series of aminoindanes with the intention to invent an anti-Parkinsonian drug. Although none of the resulting substances antagonized Parkinsonian-like symptoms (in a model of oxotremorine-induced tremors) nor showed any dopaminergic properties in mice, some of the molecules showed monoamine oxidase (MAO) inhibition and analgesic activity. Therefore, they investigated molecules with higher MAO-inhibiting potential *in vivo* and identified a candidate molecule N-methyl-5-methoxy-1-indanamine in mice. The authors concluded that the size of amine substituent and position of methoxyl substitution are most important for their biological activity (22).

Kalir et al. (23) examined the inhibitory action of substances containing aminoindanes on brain mitochondrial MAO type A and B, to ascertain MAO B inhibitors’ anti-Parkinsonian potential. Two irreversible, selective-type MAO B inhibitors were identified: AGN-1133 (*N*-methyl*-N-*2-propynyl-1-indanamine hydrochloride) and AGN-1135 (*N*-propargyl-1R-aminoindane). AGN-1135 showed greater selectivity *in vitro* and *in vivo*, with no central nervous system, cardiovascular, or sympathomimetic effects and was eventually patented as a Parkinson’s disease treatment (US patent no. 5457133A; US patent no. 5387612A; US patent no. 5453446A), known as rasagiline (24). The key difference between rasagiline and its analog selegiline is that rasagiline’s major metabolite is aminoindane, whereas selegiline metabolizes to l-amphetamine and l-methamphetamine (24, 25). Therefore, no AMPH-like adverse effects are seen after rasagiline.

### Aminoindanes–A Unique Drug Class with Entactogenic Properties

Contemporary research has focused on the psychoactive effects of substituted 2-AIs (9–13, 26–31). In their earlier work, Nichols et al. (32) proposed a new class of therapeutic psychoactive substances “entactogens,” which were neither hallucinogens nor psychostimulants; instead, they facilitated communication and introspection, and were argued to be valuable agents in psychotherapy and potentially powerful tools for understanding the neurochemistry of emotion (27). To begin with, entactogens included MDMA, MDA, and 3,4-methylenedioxy*-N-*ethylamphetamine (MDEA), and later, related novel compounds such as 1,3-benzodioxolyl*-N-*methylbutanamine (MBDB) and MDAI were synthesized. Since MDMA and its analogs (MDA, MDEA) had been widely abused by recreational drug users and serotonergic neurotoxicity was identified, Nichols et al. refocused on the preparation of non-neurotoxic analogs of MDMA. The result was the description, for the first time, of these novel “entactogenic” compounds (27, 32).

Nichols et al. (9) described effects of MDAI on catecholamines and serotonin (5-HT), measured metabolite levels, and determined the affinity (*K*_D_) and number of binding sites (*B*_max_) for 5-HT transporter (SERT) (in rat brain cortical resp. hippocampal homogenates) measured 1 week after subcutaneous (s.c.) administration of 40 mg/kg MDAI. After 1 week of recovery, there were no significant changes in levels of any of the measured neurotransmitters or SERT compared with controls; by contrast, significant reductions in the neurotransmitter levels and SERT were induced by MDMA. No changes in *K*_D_ and *B*_max_ were observed, indicating no detectable 5-HT neurotoxicity or 5-HT terminal degeneration. However, drug discrimination experiments with MDMA-trained rats showed that MDAI fully substitutes for MDMA and that MDAI and MDMAI were observed to completely substitute for another MDMA-like drug MBDB (10). It was concluded that both drugs have MDMA-like behavioral pharmacology but without lasting 5-HT neurotoxicity following an acute, very high dose. However, the effects of chronic administration of MDAI (most drugs, whether for medical or recreational purposes are taken on multiple occasions) were not investigated until much later, and research on the chronic effects of aminoindanes, including MDAI, is still lacking.

A study on *in vitro* monoamine reuptake inhibition (using rats’ synaptosomes) identified MDAI as a highly potent inhibitor of 5-HT and dopamine (DA) reuptake rather than causing non-vesicular DA release. 5-IAI and MMAI were subsequently evaluated, both of them increased non-vesicular release of 5-HT, DA, and norepinephrine (NE), but MMAI had 100- and 50-fold selectivity for 5-HT over DA and NE uptake inhibition, indicating that it is a very selective serotonergic releaser (28). In the monoamine reuptake transporter inhibition test performed on HEK 293 (human embryonic kidney 293) cells, MDAI’s ability to preferentially inhibit the NE transporter (NET) and SERT over the DA transporter (DAT) was confirmed, with an approximately twofold lower potency compared with MDMA. The other aminoindane tested, 5-IAI, showed a similar pattern/ratio of inhibitory action at NET/SERT/DAT. 2-AI selectively inhibited just NET, and for SERT and DAT it has low potency. Apart from inhibitory actions on transporter molecules, aminoindanes have been shown to cause transporter-mediated release (reverse transport) of monoamines: MDAI released 5-HT and NE, 5-IAI released 5-HT and DA, and 2-AI released NE and DA (33).

The pharmacokinetics of MDAI in Wistar rats have been described in our recently published paper (34). Tissue samples were collected after a single bolus of MDAI (10 mg/kg, s.c.) at intervals of 30, 60, 120, 240, and 480 min after administration. Separated sera, whole brains, livers, and lungs were analyzed. MDAI showed fast and high influx into the brain; the drug was accumulated in lungs where the concentration exceeded the concentration in the brain by approximately 30% (~30 vs. 18 µg/g, respectively) indicating its high-lipid solubility (34). When compared with s.c. MDMA in Sprague-Dawley rats (35), the kinetic profile of MDAI is much faster and its storage profile is similar to PMMA or 2C-B (36, 37). These results can be associated with potential selective MDAI neurotoxicity, exacerbated by combination with other drugs (6).

### Subjective Effects and Acute Behavioral Studies

Very little is known about acute behavioral effects of aminoindanes in animal studies. We described acute behavior in Wistar rats after MDAI administration. Three different s.c. doses of MDAI (5, 10, 20, and 40 mg/kg) administered (at two testing onsets 15 respectively 60 min) prior to open field test (OFT) and prepulse inhibition test (PPI) were examined to evaluate effects on locomotor activity and sensorimotor gating. At all doses used, MDAI showed a disruptive effect on sensorimotor gating and, most evidently, at testing onset 15 min. The same disruptive effect on PPI can be seen after MDMA, AMPH or other psychoactive drugs (37), and it is related to changes in sensory filtering of information due to manipulation with DA and 5-HT levels in brain (38). These changes may alter information processing and induce a schizophrenic state (39). MDAI increased trajectory length in a dose-related manner, but not dramatically. MDAI has short-acting, slightly stimulatory and anxiolytic effects (34). In another animal model, in Swiss-Webster mice, Gatch et al. (40) examined the effect of MDAI [1, 3, 10, and 30 mg/kg; administered intraperitoneally (i.p.)] on locomotor activity. Lower MDAI doses produced a rapid onset of locomotor depression and at higher doses, a slower onset of locomotor stimulation was observed, but it was longer lasting. These findings suggest that although MDAI affects DA and stimulation, this is not a strong effect. This can lead users to combine MDAI with other drugs with stimulatory potency.

Since no clinical trial has yet been performed in humans with recreational aminoindanes, information about subjective effects and health risks comes from subjective personal experiences shared on drug website platforms, wikis, and discussion fora. Based on users’ reports on PsychonautWiki, Erowid, Drugs-Forum, and Bluelight, MDAI and 5-IAI effects are mainly euphoria, empathy, stimulation (not the case with MDAI), and cognitive enhancement. The adverse effects described by users include dehydration, increased perspiration, anxiety, depression, panic attacks, and tachycardia. Several routes of administration have been reported from insufflation, oral ingestion to rectal application. The latter has the fastest onset of effects. Smoking and injecting have not been described (6, 41, 42). The onset of subjective psychoactive effects is reported to be around 30 min and their peak varies from 45 min up to 3 h after being taken orally. The wide time-window for peak effects after oral use could be caused by different product purities (6, 43): administration routes and factors influencing absorption (e.g., with oral consumption, food in the digestive tract). Users’ “recommended” dose for a mild MDAI effect is 100–150 mg, for 2-AI it is 10–20 mg orally (43, 44). The doses of 5-IAI in trip reports are approximately 100 mg orally for a mild effect (45).

### Toxicity and Health Risks

Palenicek et al. (34) examined an acute toxicity including median lethal dose (LD_50_). The highest dose of MDAI (40 mg/kg) showed 50% greater locomotion activity compared with 20 mg/kg during the onset of its action; however, animals rapidly began to hyperventilate and showed signs of serotonin syndrome (intense perspiration, copious salivation, and seizures). In total, 100% of the rats died within 15 min of administration. This was unexpected, since Nichols et al. (9) had previously used this dose and route, and did not report adverse effects or fatalities. While for s.c. administration the LD_50_ was 28.3 mg/kg and i.v. 35 mg/kg, for oral administration all rats survived 40 mg/kg (34). The autopsy and histologic evaluation of tissues of deceased animals confirmed serotonin syndrome as a causal factor in death, with disseminated intravascular coagulopathy and brain edema implicated. Gatch et al. (40) tested MDAI at 100 mg/kg, with a similar outcome to Palenicek et al. (34): this dose was lethal for all mice. Experiments on thermoregulation clearly showed that MDAI dramatically increased body temperature accompanied by profound perspiration, particularly when administered to rats housed in groups. This, along with the other findings from this study, suggests a potentially higher risk of serotonergic toxicity when the drug is used by humans in settings such as clubs or rave/dance parties, where ambient temperatures are increased due to crowding.

Since recreational users take these ecstasy-like drugs frequently in the environment of rave/dance parties for euphoric and entactogenic effects but also to enhance their abilities to dance for long periods, many users desire stimulatory effects. However, in the case of aminoindanes, where primary activity is on the 5-HT system, stimulation is limited. This often leads users to consume aminoindanes in larger doses (to increase the DA release) or in drug cocktails with stimulants such as AMPH, cocaine, or MDMA to potentiate the stimulatory properties of the drug. In these combinations, when 5-HT-ergic substances potentiate DA-ergic substances, an unexpected neurotoxicity and cardiotoxicity may occur (6, 31). Tormey and Moore (46) reported a steady increase in deaths in Ireland from 9 in 2004 to 47 in 2009 from the drug category that includes NPSs (but also includes substances such as solvents); by contrast, their data for cocaine, stimulants, and hallucinogen deaths suggest a peak in 2007, followed by a decline (which would be accounted for if “classic” drugs were being replaced by NPSs). MDAI has been related to renal failure, acute respiratory distress syndrome, hepatic failure, and increased risk of primary pulmonary hypertension or valvular heart disease (47). Furthermore, MDAI-related deaths have been reported: a 17-year-old woman died of cardiac arrest with postmortem toxicological tests detecting MDAI at a concentration of 26.3 mg/L and an ethanol concentration of 14 mg/dL. No other drugs or metabolites were detected. In the other two deaths (men aged 35 and 28), the postmortem toxicology showed MDAI along with AMPHs, MDMA, lignocaine, etc., and ethanol (6). A 27-year-old man was successfully resuscitated by paramedics but died in hospital the following day, with edema of the brain and lungs, aspiration pneumonia, blood-congested internal organs (and MDAI concentrations of 38 µg/L in peripheral blood and 1800 µg/L in urine 6 h before death) (48). Two 5-IAI and 2-AI fatalities were reported between 2010 and 2012, with one case each (49).

### Legal Status

At the time of writing, only a few aminoindanes are controlled in some parts of the EU. 2-AI is controlled in Croatia, Denmark, Estonia, Finland, Hungary, Lithuania, Poland, and Portugal. MDAI is controlled in Cyprus, Czech Republic, Denmark, Estonia, Finland, Hungary, Italy, Lithuania, Portugal, and Sweden. 5-IAI is controlled in Finland, Hungary, Lithuania, and Portugal. For instance, the UK has not specifically restricted aminoindanes yet (4).

## Conclusion

Although there are some existing studies focusing on MDAI, more research should be performed on the behavioral effects and toxicity of this substance. As we have shown in this review, fatal intoxications connected with MDAI have been reported and animal studies provided evidence of its potentially deadly toxicity due to serotonin syndrome. Furthermore, there is lack of information about toxicity, pharmacokinetics, and behavioral effects of the other aminoindanes. An important issue is also the legal status of these substances, since just a few EU countries control aminoindanes. This may increase the probability of their recreational use and, in turn, the incidence of acute toxicity.

## Author Contributions

NP contributed to the writing of the paper, searched for relevant literature, and composed the main idea for the study. RH and TP discussed the content of manuscript, added comments and critical feedback, and contributed to the final version of the paper.

## Conflict of Interest Statement

The authors declare that the research was conducted in the absence of any commercial or financial relationships that could be construed as a potential conflict of interest.

## Footnotes

^1^https://www.erowid.org/.

^2^https://psychonautwiki.org/.

^3^http://www.bluelight.org/.

^4^https://drugs-forum.com/.

^5^http://www.emcdda.europa.eu/themes/new-drugs/early-warning.

^6^https://www.unodc.org/LSS/Home/NPS.
